# Constipation a Cause of Catarrh

**Published:** 1889-02

**Authors:** N. R. Gordon

**Affiliations:** Springfield, Illinois


### CONSTIPATION A CAUSE OF CATARRH.

BY N. R. GORDON, M. D., SPRINGFIELD, ILLINOIS.

It is desirable to call the attention of the profession to a condition of the digestive system, viz.: *Constipation as a cause of naso-pharyngeal disease.* It is true, constipation is only a symptom, but the condition which the term expresses is so well understood, that we shall use the word in its common significance.

Naso-pharyngeal inflammation is found in some degree in a large number of individuals, and, likewise, do we find constipation, in some degree, in the same class of individuals. The fact that an individual will contract a cold upon slight exposure, or, indeed, sometimes without an exposure, leads us to believe that a common "cold in the head" depends more for its existence upon the condition of the system than upon the exposure. The exposure is only an exciting cause, while the cold is an acute naso-pharyngitis engrafted upon a deranged condition of the system.

The writer is unable to give statistics showing the percentage of naso-pharyngitis due to constipation, but, perhaps, 90 per centum would not be an extravagant estimate. Constipation, as a causative factor in catarrh, bears a certain relation to the severity of the disease. If the former is obstinate, the latter is also, and only yields to treatment when the former is relieved.

A person may undergo a score of exposures, when in good health, and not take cold, but the same exposure, when the least unwell, results in severe cold in the head. Here the exposure, which may be a draught of air, is the exciting cause, a small thing, but it is sufficient to disturb the equilibrium of the circulation; the exposure is not the cause of the cold, any more than the fuse which ignites the powder, is the cause of an explosion. The actual cause of a cold is found in the majority of instances in the condition of the digestive and secretory organs, which are in a state of functional inactivity, known as constipation. When this condition exists, the subject has cold feet, headache, and is liable to frequent colds. There is diminished nerve power, as is shown by the general pallor of the face, and a peculiar nervous, or irritable, condition of the entire body. While in this state the system is very sensitive to external influences, such as a draft of air, damp feet, and any little exposure will produce a shock of the nervous system, resulting in acute rhinitis. Knowing the cause of acute rhinitis, it is very easy to understand how repeated attacks will develop into chronic naso-pharyngitis.

The inactivity of the digestive organs is due to various causes, as is well known to all physicians. Among other influences which induce constipation, and one which is commonly neglected, is the form of diet, both as to quantity and quality. By the form of diet is meant consumption of large quantities of a rich and highly-seasoned food, which has the effect of impairing the activity of the bowels and overloading them with indigestible matter. A very full meal of well-cooked, plain food, is digested with greater ease than a moderate meal of rich and highly-seasoned food. When more food is consumed than the system requires, the excess of ingesta must be eliminated at the expense of the vital forces, and when this ingesta is composed largely of what is known as rich and highly-seasoned food, flavored with spices, condiments, etc., which are for the delight of the palate, rather than nutrition, it only adds to the surfeit of material to be excreted by the already overtaxed organs of excretion. The character and quality of food is a matter of great importance.

Foods are largely solids, and are mild and bland in character; they do not afford sufficient stimulation to the bowels. For instance, wheaten flour is reduced to an impalpable powder, and deprived of much of its nutritive properties. Pastries, sweet-meats, in infinite variety, rich, and indigestible, are consumed in large quantities; ice-cream and candies are extensively used. The increase in the annual consumption of sugar is simply enormous, and has much to do with the prevalence of catarrh.

The matter of diet, common as it is, should not be overlooked in the treatment of naso-pharyngeal disease. The air we breathe, the food we eat, and the water we drink, are the pillars of sustenance, and if we exercised as much care as to the fitness of food as we did the purity of the air we breathe, and limit our drinks to the form which nature gives us, there would not only be much less of the disease in question, but all other diseases would be less frequent.

In conclusion, I wish to emphasize the idea that the food, as of kind, variety, and quantity, is responsible, in a great measure, for the functional inactivity of the digestive system, and consequently the direct cause in our naso-pharyngeal affections, as well as many other diseases, and that the common exposures, such as a draught of air, or damp feet, and the thousand and one petty influences that are said to produce a cold, are only exciting causes, and as such are responsible for the production of acute rhinitis.

